# The Landscape of Geriatric Fellow Scholarly Activity Participation: Findings From a National Survey of Program Directors

**DOI:** 10.7759/cureus.47989

**Published:** 2023-10-30

**Authors:** Sadaf A Milani, Adeeb Ahmed, Shilpa Rajagopal, Mukaila Raji

**Affiliations:** 1 Department of Epidemiology, University of Texas Medical Branch, Galveston, USA; 2 Department of Internal Medicine, University of Texas Medical Branch, Galveston, USA; 3 Division of Geriatrics and Palliative Medicine, Department of Internal Medicine, University of Texas Medical Branch, Galveston, USA

**Keywords:** attitudes toward research, research training, geriatric education, scholarly activity, geriatric fellowship

## Abstract

Introduction

As the US population continues to age, there is a critical need for geriatricians to be trained and engaged in research to inform high-quality care for older adults. Our objective was to understand the extent, type, barriers, and facilitators of research training and the attitudes toward research training and scholarly activity among Accreditation Council for Graduate Medical Education (ACGME)-accredited US geriatric fellowship programs.

Methods

We conducted a cross-sectional survey of geriatric fellowship program directors from September to November 2022. Surveys assessing program characteristics, requirements for scholarly activity, director demographics, and director attitudes toward scholarly activity were distributed via email. We used descriptive statistics to assess fellowship scholarly activity requirements, facilitators, and perceived barriers.

Results

The survey response rate was 35.3% (41/116 programs). Most programs (82.9%) required participation in scholarly activity and provided protected time (73.2%). Definitions of scholarly activities greatly differed among programs. The most common scholarly activity requirements included participation in a scholarly project (70.7%) or local presentation (46.3%). The short duration of fellowship was the most common major barrier, reported by 70.7% of directors. Lastly, 34.1% of directors indicated satisfaction with the quality of research training provided, while 65.9% of directors reported satisfaction with the opportunities provided to participate in scholarly activities.

Conclusions

Overall, program requirements, facilitators, and perceived barriers to scholarly activity were heterogeneous among US geriatric program directors. Additionally, only about one-third of directors were satisfied with the research training provided. Our future work will compare the attitudes and reported barriers/facilitators of program director and fellow participants toward participation in scholarly activity.

## Introduction

As the number of older adults continues to increase in the United States (US), there is a greater demand for geriatricians to help address the unique care challenges that accompany aging [1]. Concurrently, there exists a serious need for geriatric medicine and gerontology research to holistically treat and support older adult patient populations. However, despite these observations, studies have demonstrated a shortage of geriatric providers and limited growth in geriatric training programs [2]. A 50% increase in geriatrician demand is expected between 2018 and 2030, yet the current number of certified geriatricians across the US is 7,123 providers, with approximately 253 internal and family medicine residents joining a geriatric fellowship in the year 2020-2021 [3,4]. There is also an expected shortage of academic geriatricians who educate future medical providers and lead research aimed at improving care for older adults [5].

The history of formalized geriatrics training in the US is relatively new as such programs gained traction following the creation of the National Institute on Aging in 1974 and the opening of the first Veterans Administration’s Geriatric Research, Education, and Clinical Centers in 1976 [6,7]. In 1995, the geriatrics fellowship was shortened to a one-year program length as an incentive to expand enrollment, with the change implemented in 1998 [6-9]. In recent years amid the landscape of one-year fellowship programs, a review of the existing literature demonstrated that there is minimal research specifically discussing scholarly output among geriatric fellows. A national survey of fellowship-trained geriatricians between 1990 and 1998 found that 13% of respondents identified research opportunities as the most important factor in pursuing geriatrics, and 17% of respondents expressed a need for greater research education and support, especially among those interested in academic medicine [10,11]. With the rise of the “competency-based medical education movement” and prioritization of scholarly and research output in residency, it is important to understand how geriatric training programs can better support fellows’ learning in these areas [12].

We conducted an online survey of current programs of Accreditation Council for Graduate Medical Education (ACGME)-accredited geriatric programs. Our objective was to understand the extent, type, barriers, and facilitators of research training and the attitudes toward research training and scholarly activity among ACGME-accredited US geriatric fellowship program directors (PDs). These findings can provide valuable information to strengthen geriatric fellowship research participation, improve residency/fellowship program structuring, and ultimately increase the amount of high-quality geriatrics-focused research.

## Materials and methods

Survey

We conducted a cross-sectional national survey of current ACGME-accredited geriatric fellowship (internal medicine) PDs. We used a survey designed by Abramson et al. [13] to assess scholarly activity training among pediatric fellows and pediatric PDs and adapted it for geriatric fellows and PDs with permission from the authors. We updated the survey wording to elicit information on fellows and fellowships rather than residents and residency. We also slightly edited the survey to reflect the geriatric fellowship experience, given that it is one year in length. We distributed surveys online, via email, to 116 PDs from September to November 2022. This survey was reviewed by the University of Texas Medical Branch Institutional Review Board and determined to be exempt (IRB# 22-0221).

Our survey (Table 1) included 18 items that assessed program characteristics, program definitions and requirements for scholarly activity, resources available to support scholarly activity, perceived barriers to scholarly activity, PD beliefs toward scholarly activity and PD satisfaction with the research training, and opportunities to provide research/scholarly activities to fellows. Questions were mostly closed-ended; however, throughout the survey, we asked PDs if they had anything additional that they would like to share about each topic.

**Table 1:** Survey for program directors

Survey for Program Directors
*1. How many fellows are currently in your program?*
*2. Does your fellowship program have a second- or third-year option?*
a. Yes, second year
b. Yes, second and third year
c. No
*3. How would you describe your fellowship program setting? Please select all that apply.*
a. University-affiliated
b. Community-based
c. Military
d. Veterans Affairs (VA)
e. Other
*4. In what region of the United States is your program located?*
a. Northeast (Connecticut, Maine, Massachusetts, New Hampshire, New Jersey, New York, Rhode Island, Pennsylvania, Vermont)
b. Midwest (Iowa, Indiana, Illinois, Kansas, Michigan, Minnesota, Missouri, Nebraska, North Dakota, Ohio, South Dakota, Wisconsin)
c. South (Alabama, Arkansans, District of Columbia, Delaware, Florida, Georgia, Kentucky, Louisiana, Maryland, Mississippi, North Carolina, Oklahoma, South Carolina, Tennessee, Texas, Virginia, West Virginia, Puerto Rico)
d. West (Alaska, Arizona, California, Colorado, Hawaii, Idaho, Montana, New Mexico, Nevada, Oregon, Washington, Wyoming)
*5. How does your program define scholarly activity? Please select all that apply.*
a. Original research studies (basic, clinical, health services, education, etc.)
b. Systematic literature review or meta-analysis
c. Case reports or case series (with references)
d. Book chapter
e. Quality improvement projects
f. Advocacy projects
g. Curriculum development
h. Local conference/workshop presentation to residents and/or medical students
i. Grand rounds presentation
j. Regional or national workshop presentation
k. Other
*6. Is participation in scholarly activity a requirement for graduation?*
a. Yes
b. No
*7. What are the minimum requirements for scholarly activity in your program? Please select all that apply.*
a. Participation in a scholarly project
b. Submission of a manuscript to a journal
c. Submission of a written report to the fellowship program
d. Submission of an institutional review board (IRB) application
e. Local presentation
f. Regional presentation
g. National presentation
h. Other
*8. What resources are available in your program to support scholarly activity? Please select all that apply.*
a. A scholarship review committee
b. A research director
c. A statistician
d. Sufficient number of faculty mentors for scholarly activity
e. A research track within your program
f. A special training pathway such as an Accelerated Research Pathway or Integrated Research Pathway
g. A research curriculum
h. Opportunities for fellows to present their work-in-progress
i. A research day or other venue for fellows to present their work
j. Awarding of a prize for fellow scholarly accomplishments
k. Protected time to conduct scholarly activity
l. Funding to conduct scholarly activity
m. Funding to present accepted work at a conference
n. Access to editing services
o. Other
*9. Do fellows have protected time to do scholarly activity during their fellowship training?*
a. Yes (if yes, how much time do they have?)
b. No
*10. Please rate your level of agreement with the following statements: (Scale: strongly agree, agree, neutral, disagree, strongly disagree)*
a. Understanding the principles of research is important for a career in geriatrics
b. All training programs should have a research curriculum
c. All fellows should participate in a scholarly activity project
d. All fellows should have protected time to conduct scholarly activity
*11. Is there anything else you would like to share about your beliefs toward scholarly activity for geriatric fellows?*
*12. How much of a barrier is each of the following for fellows to participate in scholarly activity during fellowship: (Scale: major barrier, minor barrier, not a barrier)*
a. Lack of time for fellows to participate in scholarly activity
b. Lack of training for fellows to conduct scholarly activity
c. Lack of a research curriculum
d. Lack of faculty mentorship to conduct scholarly activity
e. Lack of statistical support
f. Lack of funding for fellows to conduct scholarly activity
g. Lack of funding for fellows to present scholarly activity
h. Short duration of fellowship
*13. Is there anything else you would like to share about barriers or facilitators for fellows participating in scholarly activity?*
*14. How satisfied are you with the following? (Scale: very satisfied, satisfied, neutral, not satisfied, very dissatisfied)*
a. The quality of research training you provide for fellows
b. The opportunities your program provides fellows to participate in research or scholarly activities
*15. Is there anything else you would like to share about your satisfaction with the research training and opportunities to provide in research/scholarly activities that your program provides to fellows?*
*16. Gender*
a. Man
b. Woman
c. Nonbinary
d. Transgender
e. Other
f. Prefer not to respond
*17. Ethnicity*
a. Hispanic or Latino/a
b. Not Hispanic or Latino/a
c. Prefer not to respond
*18. Race*
a. American Indian or Alaska Native
b. Asian
c. Black or African American
d. Native Hawaiian or other Pacific Islander
e. Caucasian or White
f. Multiracial

Statistical analyses

We used descriptive statistics (frequencies, means) to summarize PD responses to PD demographics, program characteristics, scholarly activity definitions and requirements, resources available to fellows, barriers toward participation in scholarly activity, PD beliefs about training, and PD satisfaction with the quality of research training provided and opportunities provided to fellows to participate in research or scholarly activity. We conducted some additional analysis examining the role of program length in barriers and satisfaction. Fisher’s exact tests were used to compare the proportion of PDs who reported the short duration of the fellowship as a barrier by program length. We also used Chi-squared tests of independence to compare satisfaction with the quality of research training provided and opportunities provided to fellows to participate in research or scholarly activity (very satisfied/satisfied versus neutral/not satisfied/very dissatisfied) by program length (one-year versus second- or third-year option). Stata 17.0 (StataCorp., College Station, TX) was used for all analyses.

## Results

Program director and program characteristics

Our survey response rate was 35.3% of ACGME-accredited geriatric fellowship PDs (41/116 programs). Most PDs identified as White (68.3%), non-Hispanic (87.8%), and female (73.2%). The mean and standard deviation (SD) of the number of fellows in the program were 2.6 and 1.9 on average, and most programs were one year in length. Almost all of the programs (90.2%) were in university settings and geographic regions across the country (Table 2).

**Table 2:** Geriatric fellowship program director demographics and program characteristics (n = 41)

Characteristics	Program directors (n = 41)
Program director characteristics	n (%)
Race	
White	28 (68.3%)
Asian	11 (26.8%)
Other/prefer not to respond	2 (4.9%)
Ethnicity	
Not Hispanic or Latino/a	36 (87.8%)
Other/prefer not to respond	5 (21.2%)
Gender	
Man	10 (24.4%)
Woman	30 (73.2%)
Prefer not to respond	1 (2.4%)
Program characteristics	
Number of fellows, mean (SD)	2.6 (1.9)
Length of program	
1 year	23 (56.1%)
Second-year option available	9 (22.0%)
Second- and third-year options available	9 (22.0%)
Program setting	
University	37 (90.2%)
Community	13 (31.7%)
Veterans Affairs (VA)	14 (34.1%)
Region of US	
Northeast	9 (22.0%)
Midwest	12 (29.3%)
South	13 (31.7%)
West	7 (17.1%)

Scholarly activity definitions and requirements

Most of the responding programs (82.9%) reported that participation in scholarly activity was required for graduation from the fellowship program (Table 3). Definitions of scholarly activity widely varied across programs, with the most commonly reported definitions being case reports or case series (97.6%), original research studies (95.1%), and quality improvement (QI) projects (95.1%). Minimum requirements for scholarly activity also varied widely across programs, with about 70% of programs requiring participation in a scholarly project. Most programs (73.2%) reported providing fellows with protected time to conduct scholarly activity during the fellowship. The protected time ranged from one hour per week to one month of full-time research.

**Table 3:** Scholarly activity definitions and requirements reported by geriatric fellowship program directors (n = 41)

Characteristics	Program directors (n = 41)
Participation in scholarly activity required for graduation	
Yes	34 (82.9%)
No	7 (17.1%)
Program definitions of scholarly activity	
Original research studies (basic, clinical, health services, education, etc.)	39 (95.1%)
Systematic literature review or meta-analysis	34 (82.9%)
Case reports or case series	40 (97.6%)
Book chapter	34 (82.9%)
Quality improvement projects	39 (95.1%)
Advocacy projects	17 (41.5%)
Curriculum development	30 (73.2%)
Local conference/workshop presentation to residents and/or medical students	30 (73.2%)
Grand rounds presentation	33 (80.5%)
Regional/national workshop presentation	31 (75.6%)
Educational projects	1 (2.4%)
Minimum requirements for scholarly activity	
Participation in a scholarly project	29 (70.3%)
Submission of a manuscript to a journal	2 (4.9%)
Submission of a written report to the fellowship program	7 (17.1%)
Submission of an institutional review board (IRB) application	2 (4.9%)
Local presentation	19 (46.3%)
Regional presentation	4 (9.8%)
National presentation	7 (17.1%)
Abstract submission to national meeting	3 (7.3%)
Quality improvement (QI) project with a written report	1 (2.4%)
Fellows have protected time to conduct scholarly activity during the fellowship	
Yes	30 (73.2%)
No	11 (26.8%)

Resources available

The most commonly available resources included funding to present accepted work at a conference (78.0%), sufficient faculty mentors (73.2%), work-in-progress presentation opportunities (73.2%), protected time to conduct scholarly activity (65.9%), and a research day or other venue for fellows to present their work (56.1%), as shown in Table 3. Less than half of the PDs reported a research director (41.5%) or a statistician (41.5%) as available resources for fellows. The less common resources included a research curriculum (24.4%), access to editing services (14.6%), funding to conduct scholarly activity (12.2%), awarding of a prizefor fellow scholarly accomplishments (9.8%), a scholarship review committee (7.3%), a research track within the program (7.3%), and formal QI training (2.4%), as shown in Table 4.

**Table 4:** Resources available to geriatric fellows as reported by geriatric fellowship program directors (n = 41)

Resources	Program directors (n = 41)
Funding to present accepted work at a conference	32 (78%)
Sufficient number of faculty members for scholarly activity	30 (73.2%)
Opportunities for fellows to present their work-in-progress	30 (73.2%)
Protected time to conduct scholarly activity	27 (65.9%)
A research day or other venue for fellows to present their work	23 (56.1%)
A research director	17 (41.5%)
A statistician	17 (41.5%)
A research curriculum	10 (24.4%)
Access to editing services	6 (14.6%)
Funding to conduct scholarly activity	5 (12.2%)
A scholarship review committee	3 (7.3%)
A research track within your program	3 (7.3%)
Awarding of a prize for fellow scholarly accomplishments	4 (9.8%)
Formal training on quality improvement (QI)	1 (2.4%)

Barriers to participation in scholarly activity

**Figure 1:**
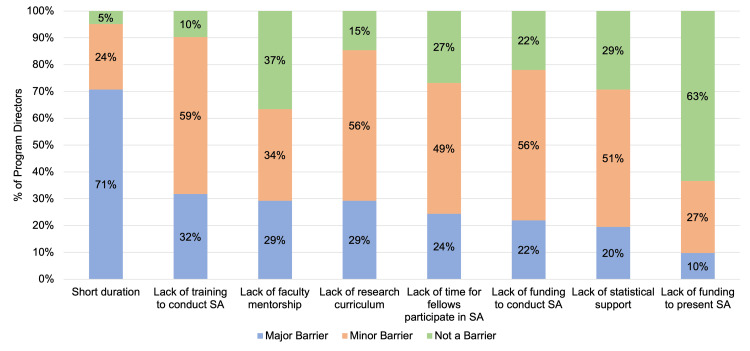
Geriatric fellowship program director reported barriers toward participation in scholarly activity (SA) during fellowship (n = 41)

The most common barrier to participating in scholarly activities reported by PDs was the short duration of the fellowship (Figure 1). This was reported as a major barrier by 71% of PDs and a minor barrier by 24% of PDs. Even among programs with second- or third-year options, the short duration of the fellowship was reported as a major barrier (60.9% of PD for one-year programs, 77.8% of PD for programs with second-year options, 88.9% of PD for programs with second- or third-year options; p = 0.616). Other commonly reported barriers include a lack of training to conduct scholarly activity and a lack of research curriculum. Lack of funding to present scholarly activity was the least common barrier, with 63% of PDs reporting that this was not a barrier.

Program director's beliefs and satisfaction with scholarly activity

**Figure 2:**
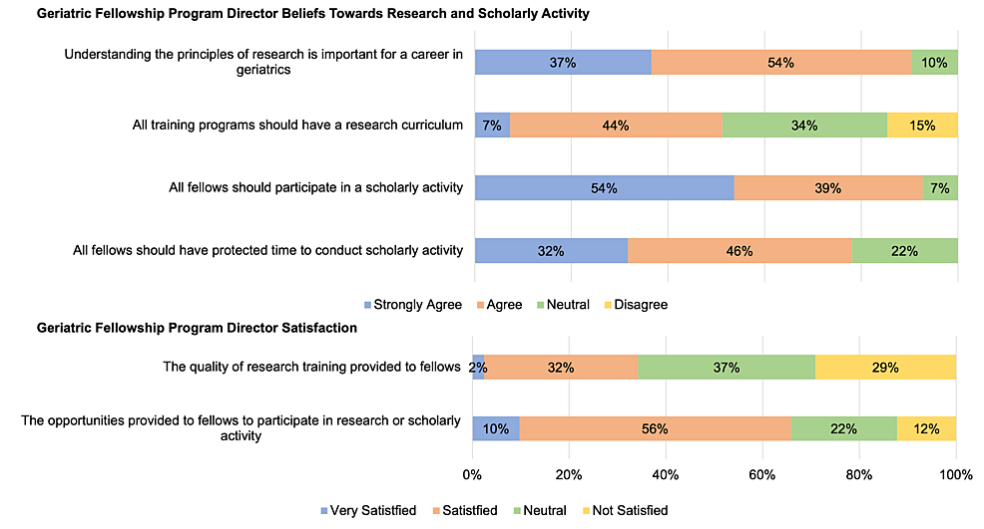
Geriatric fellowship program director's beliefs about scholarly activity and satisfaction with the quality of research training and opportunities for participation in scholarly activity (n = 41)

Most PDs (91%) agreed that understanding the principles of research is important for a career in geriatrics (Figure 2). About half of the PDs (51%) agreed that all training programs should have a research curriculum, while 34% were neutral and 15% disagreed. Most PDs also agreed that all fellows should participate in scholarly activity (93%) and should have protected time to conduct scholarly activity (78%). Only 34% of PDs were very satisfied or satisfied with the quality of research training provided to fellows, while 66% of PDs were very satisfied or satisfied with the opportunities provided to fellows to participate in research or scholarly activity.

We compared satisfaction based on program length and found no difference in satisfaction with the quality of research training (p = 0.571) or satisfaction with opportunities provided to fellows to participate in research or scholarly activity (p = 0.447) between one-year programs and the programs that had two- or three-year options.

Free-text comments from program directors

PDs were also asked to share any additional thoughts regarding barriers or facilitators for fellows participating in scholarly activity as well as general satisfaction levels and beliefs toward scholarly activity for geriatric fellows. Two themes emerged from these free-text responses: (1) the challenges of incorporating research due to program time constraints and (2) the need for expanded research-related resources, mentorship, and training opportunities for fellows. In particular, the one-year time frame was commonly cited as a significant barrier to fellows’ pursuit and completion of research projects. One program director stated that “The Graduate Medical Education funding for the ACGME year makes protected time for scholarly work challenging, and it would be helpful for fellowships to share innovative ways/best practices for protecting this time for fellows.” Another respondent noted that “scholarship activities have been modified to ensure they can be accomplished during one-year clinical fellowship program; for those who can spend more time in training, the opportunities are likely expanded (for participation and presentation, particularly at national forums).” One PD expressed that they expect fellows to submit an abstract to the American Geriatrics Society (AGS) annual meeting, but the abstract deadline presents a challenge given that most fellows have been in their positions for about four months at that point.

Additionally, PDs indicated a desire for greater funding, protected research time, and mentorship for fellows involved in scholarly activity. When describing their satisfaction levels with current research training opportunities offered, one program director indicated, “We take advantage of other resources available at our institution to supplement our fellowship - including biostatistics classes, access to research faculty in internal medicine as well as statisticians and information technology/electronic medical record database specialists (when applicable) - not all are housed under geriatric medicine.” Increased financial resources such as “funding for AGS attendance and membership” were also mentioned as potential avenues to support future research and scholarly activity participation among fellows.

## Discussion

In our national survey of current geriatric PDs, we found that program requirements, facilitators, and perceived barriers to scholarly activity were heterogeneous among US geriatric PDs. A particularly notable finding was that only about one-third of PDs were satisfied with the quality of research training their program provided to fellows. This highlights an important gap in geriatric fellowship training.

The low PD satisfaction with the quality of research training provided by programs could be explained by the short program length. Shortening the geriatric fellowship to one year increased the number of applicants and improved the percentage of program fill rates, but it reduced time for fellow research activity [8]. In a survey of 76 PDs of ACGME-accredited geriatric fellowship programs (1998-1999), 67% of geriatric PDs reported that shortening the fellowship to one year negatively affected research aspects of geriatric fellowship [8]. This poses a disadvantage to academic medicine, where fellows may not be given the training to conduct much needed, and rigorous, clinically relevant research.

Only 24% of programs reported having a research curriculum in their fellowship. This may be one strategy to improve the quality of research training provided, given the short duration of the fellowship. A scoping review of papers addressing barriers to pursuing a career in geriatrics found that limited research opportunities were cited as a barrier to pursuing a career in geriatrics in 17% of studies included [14]. Other specialties with longer years (two to four years) of training (e.g., cardiology, gastroenterology, hematology, oncology, and rheumatology) have been more successful in having a formal research training curriculum. The research curriculum in these longer-duration fellowships allows the fellows with the time and resources to undergo the training to conduct rigorous, clinically relevant research. For example, Marbach et al. described the outcomes of their implementation of a structured longitudinal research curriculum spread across the three-year cardiology fellowship training [15]. They found a substantial increase in the number of publications and the first/senior author papers during the fellowship and two years following the fellowship. Abramson et al. examined resources and barriers for scholarly activity during the three-year pediatric fellowship and found that over 90% of programs had either a departmental or division-specific research curriculum or both [16]. As the geriatrics fellowship is one year, it poses a challenge to implement a longitudinal research curriculum spread across 12 months, especially since the fellows also have clinical training obligations. One way to improve geriatrics fellowship research training would be to partner with longer-duration fellowships (e.g., cardiology, rheumatology, and hematology-oncology specialties) with a substantial proportion of geriatric patients and more established research curriculum, thus giving opportunity for potential collaborative project/publication by the subspecialty fellows and the geriatrics fellows.

This may be challenging because of the high clinical demand faced by fellows and geriatric programs. This demand will only continue to worsen as the number of older people continues to increase as the geriatric workforce capacity decreases [4,5]. In 2023, only 43% of geriatric fellowship positions were filled [17]. Geriatricians are critically needed for the care of the most vulnerable, complex, patients [18]; however, in order to provide their expertise most productively, geriatricians should also be leaders in research, QI methodology, medical education, or health care delivery [19].

In 2001, 2005, and 2010, directors of US-accredited geriatric medicine programs were asked to complete online surveys to collect data on program priorities, resources, and barriers to implementing academic geriatric programs [20-22]. These surveys have found that the number of geriatrics research faculty has not increased, and this is a commonly cited barrier to achieving their program goals [22]. Interestingly, this was not a barrier that was commonly reported by current PDs in our study. Over 70% of PDs reported that their programs had enough faculty mentors to support scholarly activity. This may be because 90% of responding PDs were affiliated with university programs.

Another strategy to improve research and scholarly activity among incoming geriatric fellows is to start having conversations, prior to their fellowship start date in July, about research projects, designing research projects, and beginning the preliminary steps (literature review, conceptual model, and potential data sources). By doing so, fellows may have an abstract ready to submit for the AGS meeting, which has a submission deadline in early December. The early conference deadline, relative to the start of the fellowship in July, was cited as a challenge for fellow research participants.

Lastly, encouraging fellows from different specialties to work together may be another strategy to increase research productivity among fellows. As aging cuts across most diseases, geriatric fellows are well suited to collaborate across disciplines. Creating a combined research training program across multiple fellowships may facilitate this collaboration. Prior work has shown this to be feasible, with the implementation of a cross-specialty QI curriculum [23]. This program resulted in 24 completed projects and improved self-reported confidence in QI domains (i.e., writing a problem statement, identifying if change leads to improvement, etc.) after completion of the program [23].

Limitations

Our study has some important limitations. The study is prone to selection bias because of our response rate of 35.3% and because most responding PDs were from university programs. PDs who participated in our survey may likely have a more favorable attitude toward scholarly activity and research as this was a research study. This may have overestimated the satisfaction in the research training provided and the belief in the importance of research for a career in geriatrics. However, our response rate is comparable with previous web-based studies of internal medicine physicians, which reported response rates ranging from 24% specifically among board-certified geriatric physicians who were also board-certified in another specialty [24] to 42.9% among internal medicine physicians who were offered incentives for their participation [25].

Another limitation of the current study is the inclusion of only the internal medicine-based geriatrics fellowship programs. In the second phase of our study, we plan to conduct a survey of geriatric fellows and PDs in family medicine-based fellowships, allowing us to compare and contrast our findings between internal medicine and family medicine programs. We also may be unable to detect nuanced differences in the attitudes toward scholarly activity and research by program characteristics as we are not sufficiently powered due to our sample size.

## Conclusions

Program requirements, facilitators, and perceived barriers to scholarly activity were heterogeneous among US geriatric PDs. The short program length was the most common barrier cited by PDs. Additionally, only one-third of PDs were satisfied with the quality of research training provided by their programs. Adopting a streamlined research curriculum could be one way to increase the quality of research training within one year of the fellowship. Improving research training and engaging more geriatricians as clinician-scientists, especially in health outcomes research, QI methodology, and implementation research, is an important step toward providing policy-and-practice-actionable data to inform the provision of evidence-based and patient-centered care to the aging population. A corresponding survey was sent to first-year geriatric fellows as a part of this study. Our future work will compare the attitudes and reported barriers/facilitators toward participation in scholarly activity between PDs and fellows.
